# Investigation of the Effect of Triacetyl Resveratrol on Epithelial‐Mesenchymal Transition in Human Lung Cancer Cell Lines

**DOI:** 10.1111/jcmm.71222

**Published:** 2026-06-25

**Authors:** Nihal Üren, Ömer Erdoğan, Ayşegül Burçin Yıldırım, Seçil Eroğlu, Mehmet Tahir Hüsunet

**Affiliations:** ^1^ Department of Medical Biology, Faculty of Medicine Gaziantep Islam Science and Technology University Gaziantep Turkey; ^2^ Department of Medical Biochemistry, Faculty of Medicine Gaziantep Islam Science and Technology University Gaziantep Turkey; ^3^ Department of Histology and Embryology, Faculty of Medicine Gaziantep Islam Science and Technology University Gaziantep Turkey; ^4^ Department of Medical Genetics, Faculty of Medicine Gaziantep Islam Science and Technology University Gaziantep Turkey

**Keywords:** A549 cell line, epithelial‐mesenchymal transition, lung cancer, triasetil resveratrol

## Abstract

Our study aimed to investigate the effects of Triacetyl resveratrol (TCRV) on epithelial‐mesenchymal transition (EMT) in human lung cancer cell lines. Different concentrations of TCRV were applied to the A549 cell line for 24 and 48 h. The IC50 value of TCRV was determined by cell viability with the CCK‐8 test. Expression levels of E‐cadherin and vimentin were analysed by immunocytochemistry and qPCR after incubation with TCRV at the determined dose. Apoptosis was assessed by acridine orange/ethidium bromide (AO/EB) staining, and possible interactions of TCRV with target proteins were evaluated by in silico molecular docking. Morphological changes were observed in A549 cells application with the IC50 dose of TCRV, showing decreased cell integrity and altered morphology compared to controls. While E‐cadherin and vimentin levels decreased in 24‐h application, an increase in both markers was observed in 48‐h application. It was determined by the AO/EBr staining method that TCRV significantly increased apoptosis and, according to the in silico molecular docking results, TCRV showed a strong binding tendency with E‐cadherin and vimentin. It was demonstrated that TCRV exhibits time‐dependent antiproliferative, apoptotic and EMT‐regulating effects in A549 cells. While significant anti‐proliferative and cytotoxic effects were observed in short‐term applications, cellular restructuring and EMT‐like processes were triggered along with resistance development in some cells in long‐term applications; however, increased stress and apoptosis were determined in the general cell population. Molecular modelling results indicate that TCRV exhibits a strong binding affinity for E‐cadherin (−7.00 kcal/mol) and vimentin (−7.70 kcal/mol) at the structural level; however, while these binding data reflect the possibility of direct protein interactions, it should not be overlooked that TCRV may play an active role in the regulation of indirect regulatory mechanisms, such as transcriptional changes.

## 1 Introduction

Lung cancer is a highly aggressive and widespread disease that was responsible for 1.8 million deaths worldwide in 2020 and is estimated to cause 2.2 million new cases. It is the leading cause of cancer death in men and the second leading cause in women after breast cancer [1]. Despite therapeutic advances, lung cancer remains the leading cause of cancer‐related deaths. Recent studies have shown that epithelial‐mesenchymal transition (EMT) is associated with malignancy in different types of cancer. It has also been reported that EMT activation in cancer cells contributes to metastasis, recurrence, or therapeutic resistance [2].

EMT is a reversible physiological chain of events in which well‐polarized epithelial cells, which are important for the formation of various tissues and organs during embryonic development, through the loss of apical–basal polarity and stable cell–cell junctions, transform into a mesenchymal‐like morphology capable of movement and migration. The cause of morphological changes occurring in cells is the suppression of epithelial markers (E‐cadherin, claudins, occludins) and the increase in mesenchymal markers (vimentin, fibronectin, N‐cadherin). Abnormal occurrence of EMT can lead to pathologies such as fibrosis and progression to cancer, while induction of EMT in various cancer cell lines is associated with increased in vitro invasion and in vivo metastasis [3]. In addition to all these, it has been reported that high tumour grades, lymph node metastases, and advanced clinical stages in epithelial solid tumours are associated with EMT [4].

Vimentin is expressed during embryonic development. It is a multifunctional protein found in connective tissue mesenchymal cells, muscles, fibroblasts, endothelial cells, macrophages, neutrophils, and lymphocytes. E‐cadherin, a cell–cell adhesion protein, plays an important role in epithelial cell adhesion and the maintenance of tissue architecture [5]. The loss of E‐cadherin expression along with the upregulation of vimentin expression is a marker indicating changes in EMT in epithelial cells [6].

During EMT, the expression of mesenchymal markers vimentin and N‐cadherin increased, while E‐cadherin, an epithelial marker that is a strong suppressor of tumour cell invasion and metastasis, was downregulated. In addition, the role of EMT in tumour formation in various different cancer types such as prostate, lung, liver, pancreatic and breast cancers has been reported [7]. Upon initiation of EMT of tumour cells, adherens junctions are disrupted, types of cadherin molecules are altered, and the cytoskeleton undergoes changes to enhance motility. E‐cadherin and vimentin, which play a role as molecular markers in the maintenance of adherens junctions and cytoskeletal remodelling, have been shown to be significantly associated with the prognosis of non‐small cell lung cancer [8].

Resveratrol (RES) (3,5,4′‐trihydroxy‐trans‐stilbene) is an anticancer, antioxidant, anti‐inflammatory polyphenolic molecule found in various plants such as grapes, soy, peanuts, and tree nuts, as well as grapes. RES and its analogs reverse EMT to inhibit metastasis and reduce chemoresistance in human tumours through different pathways and mechanisms. Increasing studies have shown that RES is preventive and therapeutic against various tumour types such as colorectal, breast and lung cancers and covers various stages of cancer development such as EMT. According to various studies, resveratrol plays an important role in cancer treatment and in preventing the initiation and progression of carcinogenesis [9, 10].

A new natural derivative of resveratrol, TCRV, has been recently discovered. TCRV is a derivative of resveratrol found in natural plants and has better pharmacological activity than RSV. Phenylalanine converts to resveratrol after 4 reactions, and resveratrol converts directly to triacetyl resveratrol. Triacetyl resveratrol, a natural compound with superior bioavailability, is known to inhibit proliferation in prostate and breast tumour cell lines [11, 12].

Molecular modelling is used to describe the reactions of molecules and predict their possible macroscopic properties. Data obtained from experiments provide information but do not reveal the mechanism of action in the biological system. These mechanisms can be studied in computer modelling using the structures of biological systems [13].

## 2 Materials and Methods

### 2.1 Cell Culture

A549 human lung cancer cell lines were cultured in a medium containing 10% FBS and 1% penicillin–streptomycin at 37°C and 5% CO_2_. The prepared media were used after being sterilized with a 0.22 μM pore diameter filter. The morphology and proliferation status of the cells were followed daily under an inverted microscope. The morphology and proliferation status of the cells were followed daily under an inverted microscope. Subculturing was done when the cells were 80%–90% confluent. TCRV was applied to A549 cell lines at concentrations of 25 μM, 50 μM, 100 μM, 200 μM, 400 μM, 800 μM, and 1.6 mM for 24 and 48 h.

### 2.2 Cytotoxicity and Cell Viability Testing

A549 human lung cancer cells were seeded in 96‐well culture plates at 1 × 10^4^ cells per well. After 24 h of incubation at 37°C and 5% CO_2_, they were exposed to increasing concentrations of TCRV for 24 and 48 h. After 24 h, 10 μL CCK‐8 was added to each well and incubated for 4 h. At the end of the period, it was taken from the incubator and the IC_50_ concentrations of TCRV were determined by measuring the absorbance intensity on a 450 nm microplate reader. The same procedure was applied to cells exposed to TCRV for 48 h. The results were taken from the device and IC_50_ values were calculated.

### 2.3 Immunocytochemistry

Cells were seeded at 5 × 10^4^ cells/well onto 8‐well glass slides designed for immunohistochemical staining and cultured for 24 h. To evaluate E‐cadherin and vimentin immunoreactivity, labeling was performed with the avidin‐biotin‐peroxidase method and a staining kit was used. Cells were incubated with E‐cadherin and Vimentin antibody for 1 night at 4°C. After the necessary washes, it was kept at +4°C for 1 night with E‐cadherin and Vimentin primary antibodies. After washing, it was incubated with biotinylated IgG (secondary antibody) and streptavidin peroxidase. It was then washed and treated with diaminobenzidine for 5 min to make immunoreactivities visible and washed with distilled water. Cells counterstained with Gill haematoxylin were washed several times with distilled water and covered with drops of water‐based sealer. Images were taken with a DS‐Ri2 model digital camera under a Nikon Ni‐U model light microscope. E‐cadherin and vimentin expression density measurements were made by performing h scoring for immunoreactivity differences from the images taken. H‐scores were calculated using a semi‐quantitative assessment of staining intensity [14]. The intensity of staining was evaluated semi‐quantitatively in NIS Elements software on a Nikon Ni‐U microscope: 0, no staining; 1, weak staining; 2, moderate staining; 3, strong staining. For each antibody, 500 cancer cells were counted in each group, and H‐scores were calculated by plotting the percentage of strongly stained cells (3), the percentage of moderately stained nuclei (2), and the percentage of weakly stained cells (1). The data obtained were evaluated statistically. A *p*‐value of < 0.05 was considered statistically significant.

### 2.4 RNA Isolation, cDNA Synthesis and qPCR Reaction

After the incubation period was completed, the cells were checked under an inverted microscope. RNA was isolated from cells in the A549 line using a total RNA isolation kit according to the procedure of the RNA isolation kit. The concentrations of each of the isolated RNA samples were measured in a microplate reader. After standardising the RNA concentration in all samples, cDNA synthesis was performed using the FIREScript RT cDNA synthesis kit according to its instructions. cDNA samples were placed in the Rotorgene real‐time‐PCR device and amplified using specific primers (Table 1) according to the qPCR kit (HOT FIREPol SolisGreen qPCR Mix) protocol. After amplification, the expression levels of E‐cadherin and vimentin genes were analysed using the ΔΔCt method.

**TABLE 1:** Primers used for RT‐PCR.

Genes	Base Sequences (5′ → 3′)	Annealing (∘C)
Beta actin	F: 5′‐CATGTACGTTGCTATCCAGGC‐3′	60°C
	R: 5′‐CTCCTTAATGTCACGCACGAT‐3′	
E‐cadherin	F: 5′‐ATTTTTCCCTCGACACCCGAT‐3′	60°C
	R: 5′‐TCCCAGGCGTAGACCAAGA‐3′	
Vimentin	F: 5′‐AGTCCACTGAGTACCGGAGAC‐3′	60°C
	R:5′‐CATTTCACGCATCTGGCGTTC‐3′	

### 2.5 Determination of Apoptotic Cells by AO/EBr Staining

To distinguish and quantify the different stages of cell death, an Acridine Orange/Ethidium Bromide (AO/EBr) dual staining assay was performed. A549 cells were seeded into 12‐well plates at a density of 5 × 10^5^ and incubated for 24 h at 37°C in a 5% CO_2_ incubator. Cells were treated with TCRV at the concentration IC₅₀ determined. After 24 and 48 h of incubation the cells were stained with a mixture of AO and EBr at a final concentration of 50 μg/mL each. The plate was incubated in the dark for 10 min. Cells were visualized under a fluorescence microscope using standard FITC and Texas Red filter sets to detect AO and EBr signals, respectively. Digital images were acquired from each fluorescence channel and overlaid using ImageJ software to assess colocalization and nuclear morphology. For the quantitative determination of apoptotic indices, a total of 500 cells per experimental group were randomly selected and manually analysed using ImageJ software. Cells were classified into three populations based on nuclear morphology and membrane permeability: (i) Viable cells, (ii) Early apoptotic cells: Yellowish fluorescence characterized by chromatin condensation or nuclear fragmentation, (iii) Late apoptotic/Necrotic cells: Predominant orange to red fluorescence due to the loss of membrane integrity. The results were expressed as the percentage of the total cell population. To evaluate differences between groups, an independent samples Student's *t*‐test was conducted. Control and TCRV (triacetyl‐treated) groups were compared at both 24‐h and 48‐h time points. The statistical analyses were conducted using both Microsoft Excel (Microsoft Corp., Redmond, WA) and Python (version 3.11) implemented in the SciPy (scipy.stats) package. Consistency between results was confirmed. The *t*‐tests were applied as two‐tailed tests under the assumption of equal variances (*p < 0.1, **p < 0.05 and ***p < 0.001).

### 2.6 In Silico Molecular Docking

The 3D structures of the triacetylresveratrol ligand were downloaded in. sdf format from the PucChem database [15]. The structure of the molecule was then saved in. pdb format using the BIOVIA Discovery Studio Visualizer program. The 3D structures of E‐cadherin (PDB ID: 1FF5) and vimentin (PDB ID: 1GK4) receptors were downloaded from the Protein Data Bank database in.pdb format [16]. The AutoDock 4.0 program was used to perform molecular docking analyses [17]. Docking analysis was performed to predict possible binding sites of TCRV ligand on the crystal structure of target receptors. AutoDockTools (ADT) was used to prepare parameters before starting the receptor and ligand molecules docking analysis. Polar hydrogen atoms in the receptor and ligand molecules were retained while nonpolar hydrogens were combined. Gasteiger loads were calculated with ADT as previously described by Ricci and Netz [18] and Nasab et al. [19]. During the molecular docking experiment, all rotatable bonds of the ligand were allowed to rotate, and then the prepared receptors and ligand were saved in PDBQT format. A grid box size of 60 × 60 × 60 Å points was set with a grid spacing of 0.375 Å. Docking studies were performed using an initial population of up to 150 individuals, with 100 genetic algorithm (GA) runs, 5 × 10^5^ energy evaluations, and a maximum of 27,000 generations. The values of 0.02 and 0.8, chosen as mutation and transition rates, respectively, were applied to the population. After 100 independent docking studies for each analysis, all possible binding modes were clustered by the program, and the binding free energy of the conformation with the best docking pose and the lowest binding free energy was ranked based on the binding free energy in kcal/mol for the selected pose of the ligands. The best docking pose obtained between the ligand and receptor using AutoDock 4.0 was analysed using BIOVIA Discovery Studio Visualizer 2016 [20].

## 3 Results

### 3.1 Cytotoxic Effects of Triacetyl Resveratrol

**FIGURE 1:**
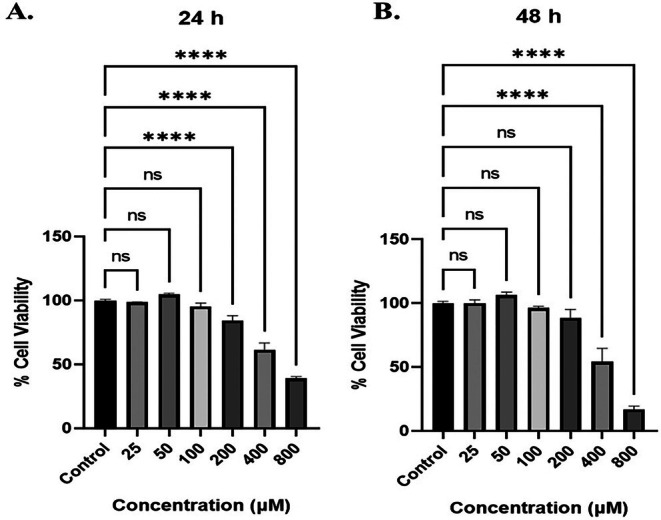
24‐h percentage viability graph of TCRV on A549 cells (A). 48‐h percentage viability graph of TCRV on A549 cells (B).

The IC_50_ value for 24 h was calculated as 473.9 μM, while the IC_50_ for 48 h was calculated as 434.3 μM. Figure 1.

### 3.2 Morphological Effects of Triacetyl Resveratrol

**FIGURE 2:**
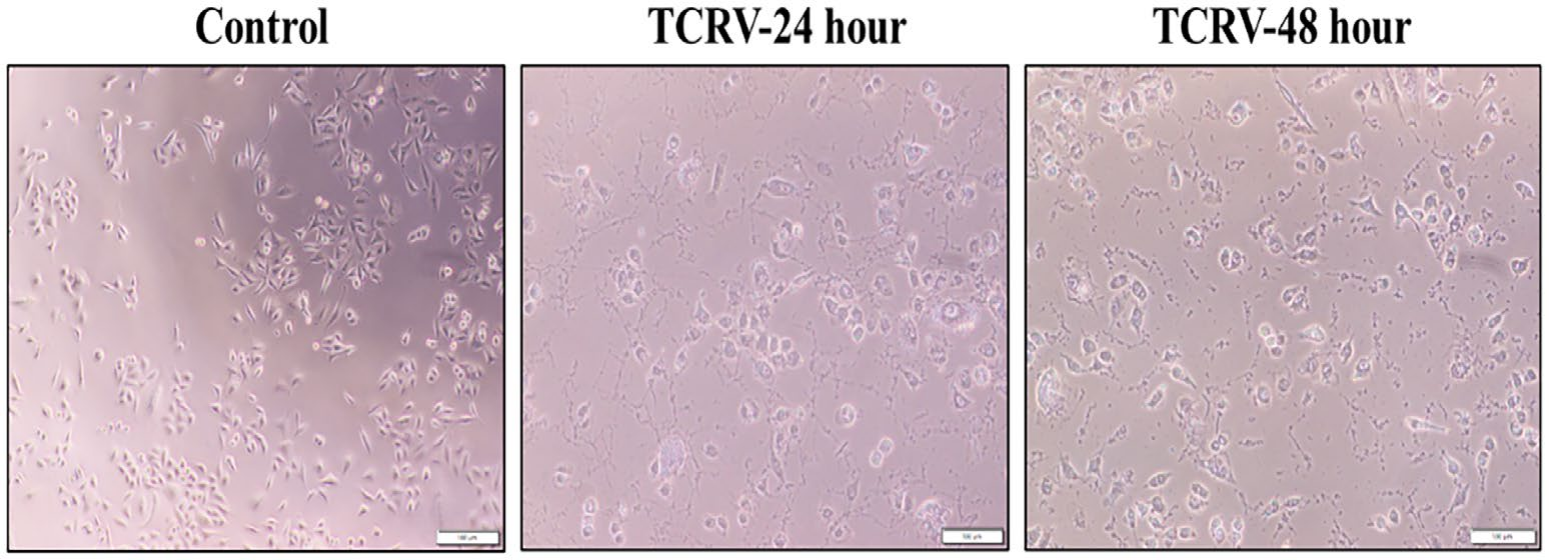
Inverted microscope images of cells in the control group and cells treated with the IC50 dose of TCRV for 24 and 48 h.

A549 human lung cancer cell line was used as the malignant cell line to evaluate the morphological effects of TCRV. Compared with the control group, significant changes were observed in the morphological images of cells administered TCRV at the determined IC_50_ doses for 24 and 48 h. It was determined that cell integrity decreased and morphological structures began to deteriorate compared to the control group. It was determined that especially after 48 h of TCRV application, cell sizes expanded and bubbles, an apoptotic marker, formed more frequently within the cells (Figure 2).

### 3.3 Effect of Triacetyl Resveratrol on Gene Expression of EMT Related Genes

**FIGURE 3:**
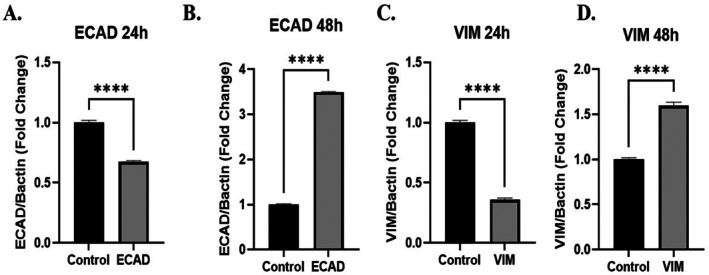
E‐cadherin expression graph of TCRV on A549 cells for 24 h (A). E‐cadherin expression graph of TCRV on A549 cells at 48 h (B). 24‐h VIM expression graph of TCRV on A549 cells (C). VIM expression graph of TCRV on A549 cells at 48 h (D).

In this study, the effects of TCRV on the gene expressions of EMT pathway were examined by QPCR. According to the results obtained, it was observed that E‐cadherin expression decreased in A549 cells exposed to TCRV for 24 h compared to the control group and E‐cadherin expression increased in the 48‐h exposure period. Similarly, it was found that Vimentin expression decreased in cells treated with TCRV for 24 h compared to the control group, while Vimentin expression increased in the 48‐h application (Figure 3).

GraphPad Prism version 8.2.0 for Windows (GraphPad Software, San Diego, California USA, www.graphpad.com) was used for statistical analysis. The normal Gaussian distribution of the findings was confirmed by the Shapiro–Wilk normality test. Comparisons of findings were made using one‐way analysis of variance (ANOVA) and Tukey's multiple comparison test. Results are presented as mean ± SD. Values of *p* < 0.05 were considered statistically significant. Significance levels are presented in the figures as follows: ****p < 0.0001, ***p < 0.001, **p < 0.01, *p < 0.05.

### 3.4 Immunocytochemistry Findings

**FIGURE 4:**
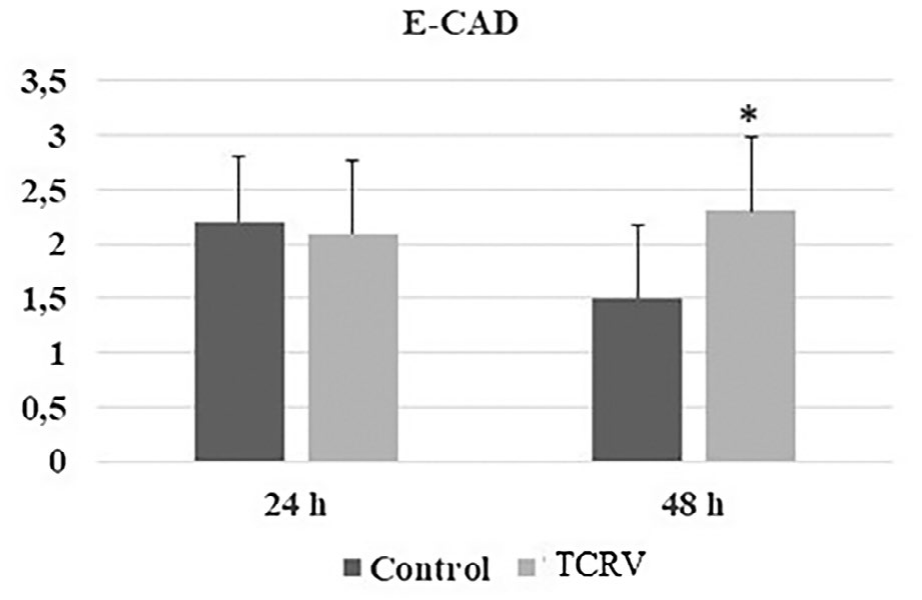
E‐cadherin expression H‐score graph at 24 and 48 h.

The significance of differences was determined by one‐way ANOVA followed by Tukey's test. Data are presented as mean ± standard deviation. When compared with the control group, there is no significant difference for 24 h (*p* = 0.07), but there is a significant increase in E‐cad expression for 48 h (*p < 0.001). According to the h scoring results, there was no significant change in E‐cad expression in the first 24 h, but E‐cad expression increased significantly at the end of the 48th hour (Figure 4).

**FIGURE 5:**
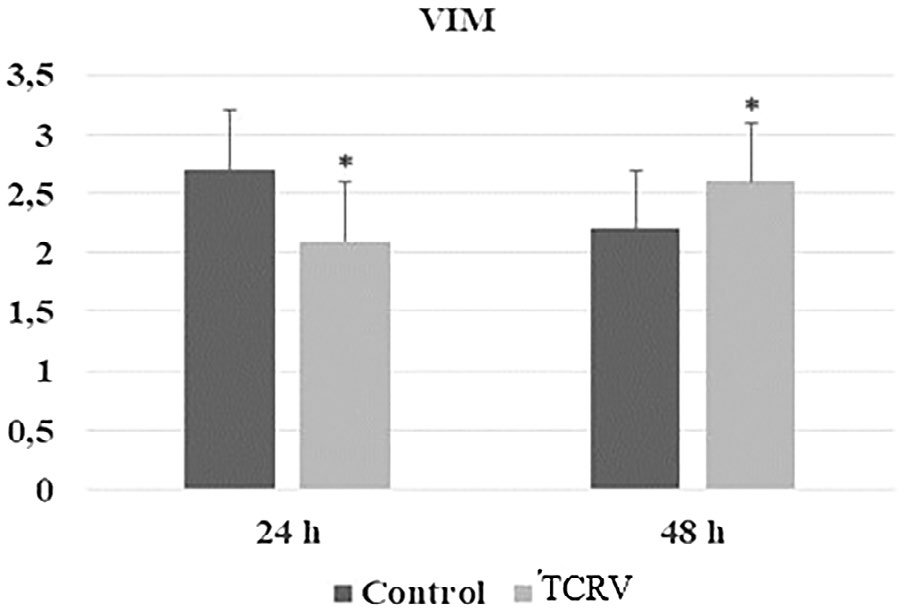
Vimentin expression H‐score graph at 24 and 48 h.

The significance of differences was determined by one‐way ANOVA followed by Tukey test. Data are presented as mean ± standard deviation. *p < 0.001 compared with the control group for 24 h and 48 h. According to h scoring results, vimentin expression decreased with TA application in the first 24 h, but increased statistically significantly at the 48th hour (Figure 5).

**FIGURE 6:**
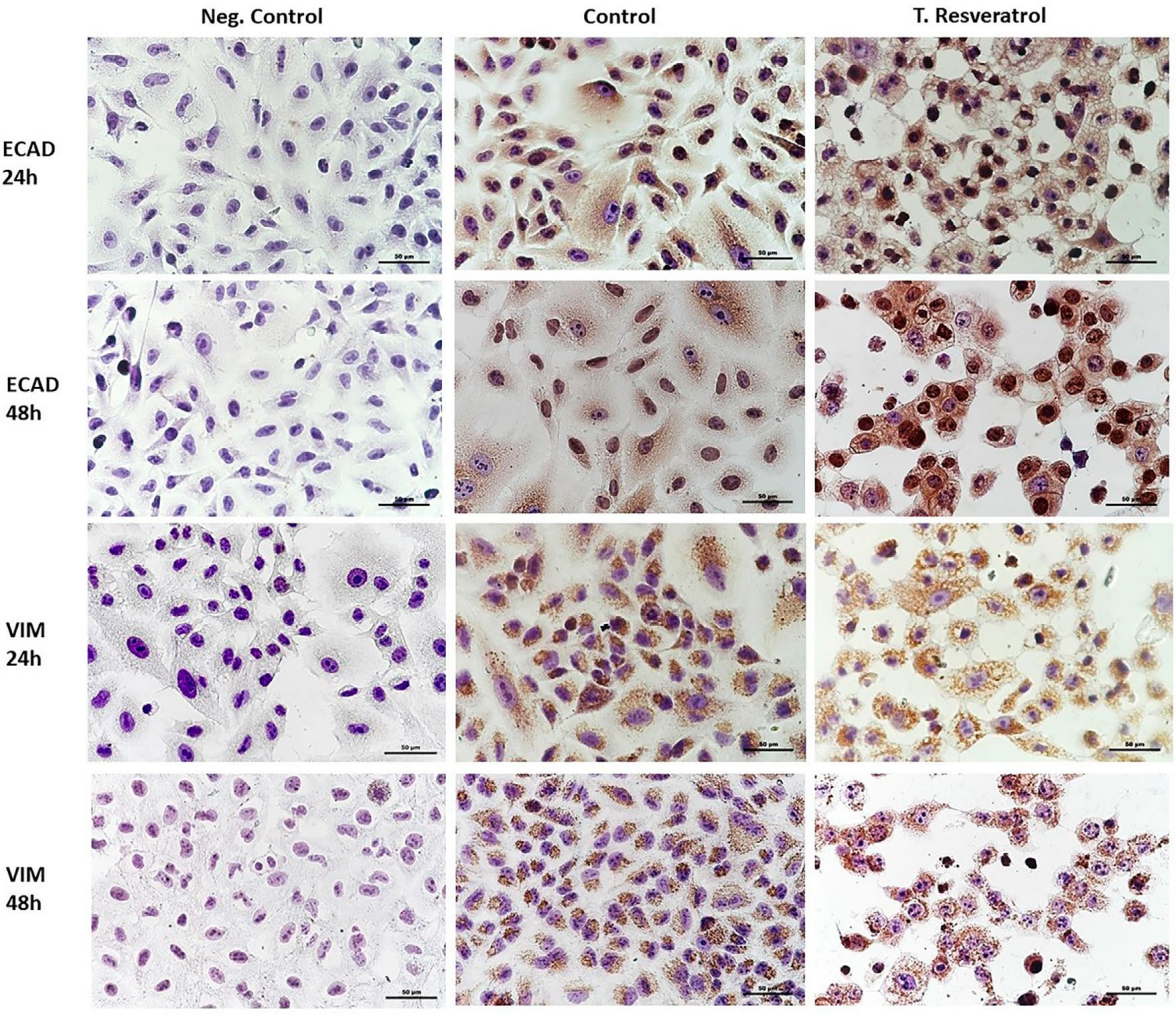
Immunocytochemical staining of E‐cadherin and Vimentin in A549 cells in all groups.

According to immunocytochemistry results, E‐cadherin and vimentin expression were examined in all groups in A549 cells. While E‐cad expression was found to be similar to the control group in 24‐h application, expression was observed to increase in 48‐h application. Vimentin expression decreased in the first 24 h with TCRV administration compared to the control group, but increased at the end of 48 h (Figure 6).

Brown areas indicate E‐cad and Vimentin positive cells. 40× magnification, scale bar 50 μm.

JASP 0.14.1.0 software was used for statistical analysis. The Kolmogorov–Smirnov test was used to determine the normal distribution of the data. Data were expressed as mean ± SD and analysed with One‐way ANOVA test and Tukey's post hoc test for parametric tests. In the analyses, *p* < 0.05 was considered significant [14].

### 3.5 AO/EBr Staining Results

**FIGURE 7:**
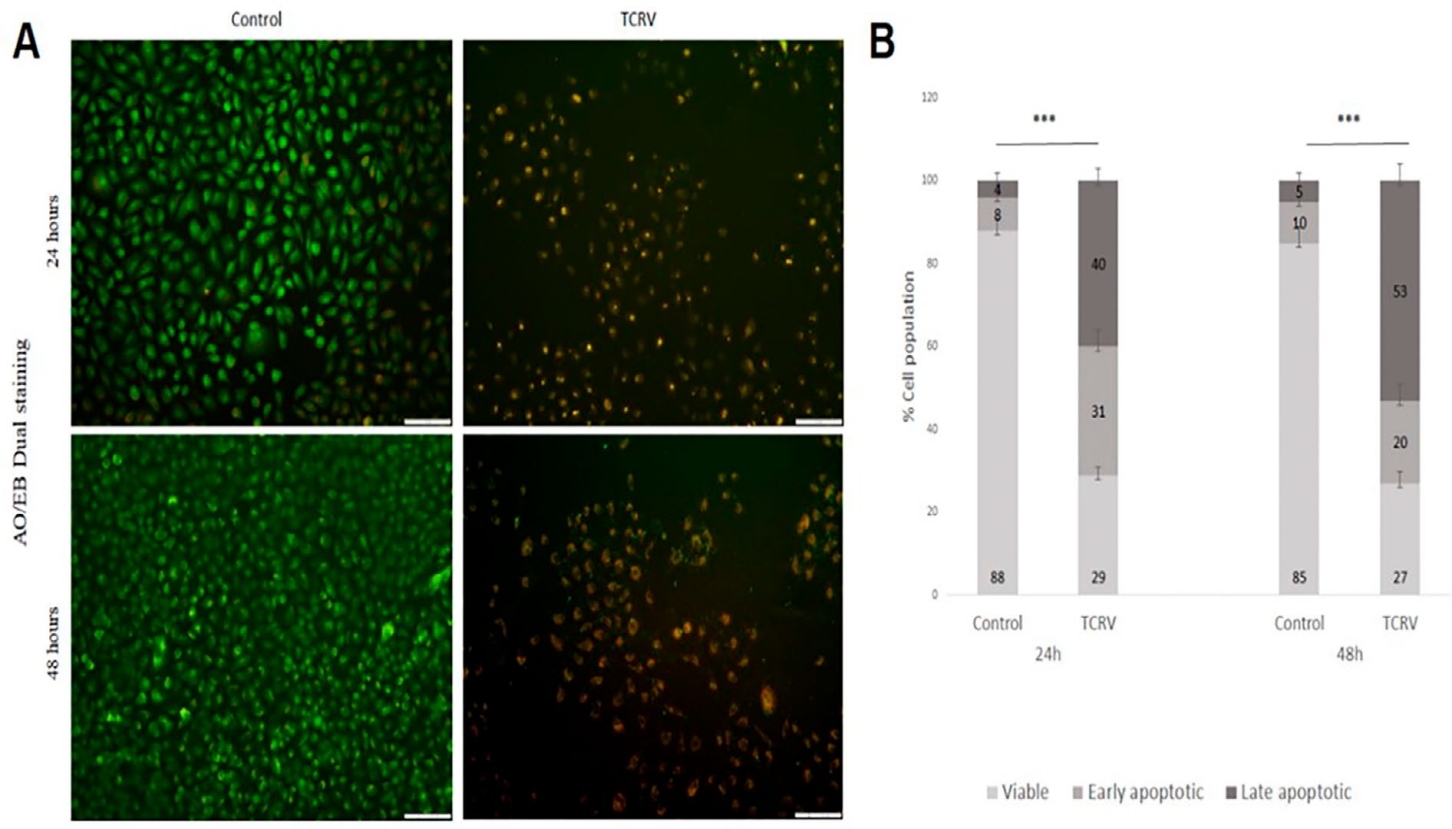
AO/EBr staining of A549 cells treated with triacetyl resveratrol for 24 and 48 h. (A) Fluorescence images of AO/EBr‐stained A549 cells showing a time‐dependent transition from early to late apoptotic stages. (B) The stacked bar graph quantifies the shift from early to late apoptotic phases between 24 h and 48 h. Distribution of viable, early, and late apoptotic cells expressed as a percentage of the total population. Results are presented as mean ± standard deviation (*n* = 3). A statistically significant difference was observed between the control and TCRV groups at both 24 and 48 h (***p < 0.0001).

AO/EBr Staining of A549 Cells treated with Triacetyl Resveratrol for 24 and 48 h (Figure 7).

### 3.6 In Silico Molecular Docking Results

**FIGURE 8:**
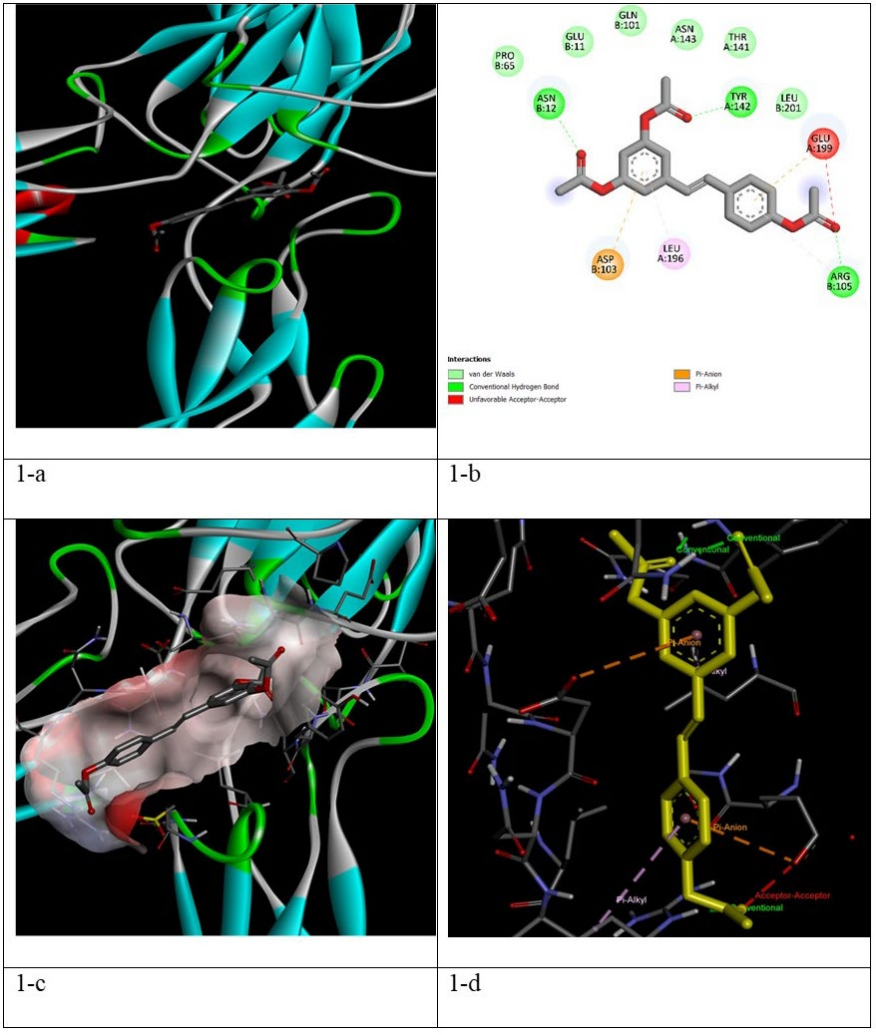
Illustration of the docking interaction between triacetylresveratrol and E‐cadherin. 1a: Best 3D Docking pose, 1b: 2D aa interaction and chemical bond types, 1c: 3D electric field interaction, 1d: Ligand interaction pose.

**FIGURE 9:**
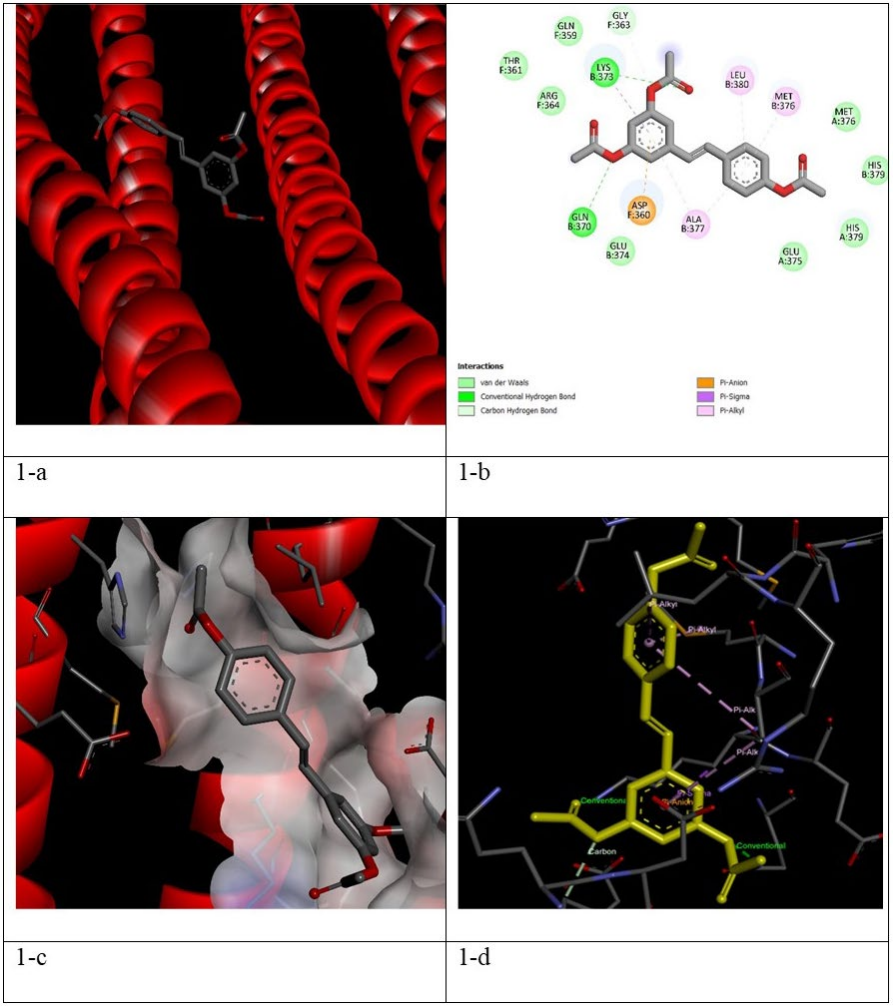
Illustration of the docking interaction between triacetylresveratrol and vimentin. 1a: Best 3D Docking pose, 1b: 2D aa interaction and chemical bond types, 1c: 3D electric field interaction, 1d: Ligand interaction pose.

The amino acid (aa) interactions of the ligands with the target molecule (E‐cadherin and vimentin) based on Gibbs free binding energies in kcal/mol and the types of chemical bonds (Figures 8 and 9) were shown. The lowest negative Gibbs free binding energy (ΔG binding) of the TCRV ligand for E‐cadherin and vimentin was determined to be −7.00 kcal/mol and −7.70 kcal/mol, respectively. While the triacetylresveratrol/E‐cadherin interaction occurred with 12 aa and 5 different chemical bonds, triacetylresveratrol/vimentin exhibited 6 different chemical bond interactions with 15 aa. Our results were considered significant because these results had stronger affinity than the threshold binding energy of −6.0 kcal/mol.

## 4 Discussion

In this study, the effect of TCRV, a natural polyphenol derivative, on epithelial‐mesenchymal transition, antiproliferative, apoptotic and molecular effects on A549 lung adenocarcinoma cells was evaluated. The findings show that TCRV reduces cell viability, activates apoptosis mechanisms and causes significant changes in cellular morphology.

According to the cytotoxicity results obtained in our study, TCRV has been shown to cause increasing cytotoxic and apoptotic effects in A549 cells depending on time. The IC50 value of TCRV on A549 cells for 24 h and 48 h were respectively calculated as 473.9 μM and 434.3 μM (Figure 1). The observed IC₅₀ values are indeed higher than those typically reported for many TCRV cytotoxicity studies [11, 21]. Several factors may contribute to this observation. Firstly, the relatively high IC₅₀ value may be related to limited aqueous solubility of TCRV at higher concentrations, which could reduce its effective bioavailability in the culture medium. Secondly, cellular uptake efficiency may be suboptimal, potentially limiting intracellular accumulation of the compound. Thirdly, metabolic instability or rapid degradation under in vitro conditions may also reduce its apparent potency [22, 23]. In morphological examinations, it was observed that cell integrity decreased in 24‐h (short‐term) application, and in 48‐h (long‐term) application, cell size enlargement and apoptotic vacuoles and structural and obvious structural deteriorations occurred (Figure 2). According to ımmunocytochemistry findings; An increase in E‐cadherin and vimentin expression levels was detected after 48 h of TCRV administration (Figures 4, 5, 6). This suggests that some cells may have undergone partial EMT‐like remodelling processes in an adaptive response to treatment, but the overall cell population exhibited high levels of cellular stress and reduced proliferative capacity.

AO/EBr staining results also support these findings. These results demonstrate that triacetyl treatment significantly enhanced apoptosis at both the 24 and 48‐h time points. While cells at 24 h primarily exhibit hallmarks of early apoptosis (yellowish fluorescence with chromatin condensation, typical of early apoptotic stages), a significant shift toward late apoptosis (orange/red fluorescence with loss of membrane integrity and nuclear fragmentation) is evident by 48 h (Figure 7). This suggests that TCRV may exhibit anticancer activity by inducing apoptotic cell death in A549 cells. Similarly, Guo and colleagues reported that resveratrol could reverse chemoresistance by inhibiting the EMT process and improving the antiproliferative effects of conventional treatments [10]. This finding supports that resveratrol and its analogs can be considered as potential therapeutic agents targeting EMT under appropriate dose and duration conditions.

At the same time, according to the results we obtained from the gene expressions of EMT related genes, it was determined that EMT markers were suppressed at the gene level in 24‐h TCRV application, and the expression of both markers increased after 48 h of exposure (Figure 3). This suggests that TCRV creates different biological responses depending on time and that a phenotypic transformation similar to restructuring or partial EMT may occur in cells within 48 h of application. In a study conducted by Fabiana Lüönd and colleagues, it was emphasized that cells in the partial EMT state exhibited varying degrees of epithelial and mesenchymal phenotypic features and expressed combinations of epithelial and mesenchymal cell markers. It has also been reported that cells undergoing partial EMT contribute to lung metastasis and chemoresistance, while full EMT cells mostly maintain the mesenchymal phenotype and cannot colonize the lungs [24]. Similarly, Fu et al. reported that ‘Triacetyl resveratrol inhibited EMT by upregulating E‐cadherin expression and suppressing N‐cadherin expression and transcription factors such as Snail, Slug and Zeb1 [25]. In addition, Xiao Chen and colleagues evaluated the pharmacological activity of RSV in A549 cells and reported that it significantly suppressed cell migration without affecting cell viability [26]. These data support the potential suppressive effect of TCRV on invasion/migration.

The decrease in EMT markers observed after 24‐h TCRV application may be due to cytotoxic stress or low‐dose apoptosis/senescence response in short‐term applications rather than direct EMT suppression. A study in the literature has shown that low‐dose RV treatment induces premature aging through ROS‐mediated DNA damage and inhibits lung cancer cell growth through this mechanism [27]. In addition, in a study conducted by Janica C. Wong and colleagues on endothelial cells, it was reported that resveratrol exhibited dose‐dependent biphasic effects; it stimulated proliferation/DNA synthesis at lower concentrations but inhibited proliferation/DNA synthesis at higher concentrations. Additionally, this study emphasized that resveratrol causes biphasic apoptotic and proliferative effects [28]. There are studies in the literature reporting that resveratrol also has direct suppressive effects on the EMT process. In particular, Heyong Wang et al. reported that resveratrol increased E‐cadherin expression in A549 cells during TGF‐1‐induced EMT, while fibronectin and vimentin were suppressed to a great extent, preventing cell adhesion, migration and invasion [29].

When we examined the molecular docking results, we observed strong hydrogen bond interactions with the triacetylresveratrol ligand and the amino acid residues ASN12, TYR142 and ARG105 aa in the E‐cadherin receptor binding motif (RBM), while van der Waals interactions were observed with the PRO65, GLU11, GLN101, ASN143, THR141 and LEU201 aa residues (Figure 8). GLU199 aa showed the second interaction, the Unfavourable Acceptor‐Acceptor bond interaction. Additionally, ASP13 and GLU199 residues were found to have Pi‐Anion bond interactions, while LEU196 and ARG105 were found to have Pi‐Alkyl bond interactions with the aa. When the docking interaction of triacetylresveratrol and vimentin was examined, hydrogen bonding with GLB370 and LYS373 aa and van der Waals interaction with GLU374, ARG364, THR361, GLN359, GLY363, MET376, HIS379 and GLU375 aa were observed (Figure 9). ASP360 aa showed Pi‐Anion interaction, while LYS373 aa showed Pi‐sigma interaction. Additionally, residues ALA377, LEU380 and MET376 exhibited Pi‐Alkyl bond interactions. The binding limit threshold energy is accepted as −6.0 kcal/mol [30]. The results were considered significant because all ligand‐receptor Gibbs free binding energies were more negative than −6.0 kcal/mol.

It should be noted that the purpose of molecular binding analysis is to predict whether an interaction between molecules is thermodynamically feasible at the structural level (e.g., enzyme inhibition), but it does not aim to directly elucidate changes in gene expression or transcriptional regulation.

Therefore, it is insufficient to interpret the decrease in E‐cadherin and vimentin expression detected 24 h later directly as TCRV binding affinity. Instead, it is important to note that changes in transcription could be caused by pathways such as Wnt/β‐catenin, TGF‐β or cytotoxic stress‐induced signalling and could be indirectly regulated by TCRV. The high binding affinities between TCRV and E‐cadherin (−7.00 kcal/mol) and between TCRV and vimentin (−7.70 kcal/mol) indicate that TCRV can structurally interact with these proteins and potentially trigger certain conformational changes in them or influence protein–protein interactions. Therefore, molecular binding results and experimental data should be evaluated as complementary pieces. While binding affinities confirm that TCRV can structurally bind to E‐cadherin and vimentin, transcriptional changes are likely more complex and do not directly stem from these interactions.

In conclusion, this study demonstrated that TCRV exhibited time‐dependent antiproliferative, apoptotic and EMT‐regulating effects in A549 lung cancer cells. While significant cytotoxic and anti‐proliferative effects were observed in short‐term applications, it was determined that cellular restructuring and EMT‐like processes were triggered along with the development of resistance in some cells in long‐term exposures; however, increased stress response and apoptosis were dominant in the general cell population. Molecular modelling results show that TCRV exhibits a strong binding affinity for E‐cadherin (−7.00 kcal/mol) and vimentin (−7.70 kcal/mol) at the structural level, which points to the possibility of direct protein–protein interactions; however, it should not be overlooked that the transcriptional changes observed in expression are more likely to be mediated by indirect regulatory mechanisms.

The fact that no comprehensive study examining the effects of TCRV on lung cancer cells has been found in the literature increases the originality and scientific potential of this research. The findings suggest that TCRV may be a potential therapeutic agent that may exhibit variable effects depending on the dose and duration of exposure. However, further studies are needed in in vivo models and different cancer subtypes to confirm this effect and to clarify its mechanisms more clearly.

## Author Contributions

**Nihal Üren:** conceptualization, methodology, investigation, formal analysis, validation, resources, project administration, funding acquisition, writing – original draft, writing – review and editing. **Ömer Erdoğan:** investigation, formal analysis, writing – review and editing. **Ayşegül Burçin Yıldırım:** investigation, formal analysis, writing – review and editing. **Seçil Eroğlu:** investigation, formal analysis, writing – review and editing. **Mehmet Tahir Hüsunet:** investigation, formal analysis, writing – review and editing.

## Disclosure

*Declaration of Generative AI Use*: The manuscript was written in English by the authors. During preparation, language‐editing tools were used to improve clarity and readability, and the final version was carefully reviewed.

## Funding

This study was supported by the Scientific Research Projects Coordination Unit (BAP) of Gaziantep Islam Science and Technology University (Project Number 2024‐GAP‐TF‐0003).

## Conflicts of Interest

The authors declare no conflicts of interest.

## Data Availability

The data that support the findings of this study are available from the corresponding author upon reasonable request.

## References

[1] A. Leiter , R. R. Veluswamy , and J. P. Wisnivesky , “The Global Burden of Lung Cancer: Current Status and Future Trends,” Nature Reviews Clinical Oncology 20, no. 9 (2023): 624–639, 10.1038/s41571-023-00798-3. 37479810

[2] Y. Otsuki , H. Saya , and Y. Arima , “Prospects for New Lung Cancer Treatments That Target EMT Signaling,” Developmental Dynamics 247, no. 3 (2018): 462–472, 10.1002/dvdy.24596. 28960588

[3] S. Jonckheere , J. Adams , D. De Groote , K. Campbell , G. Berx , and S. Goossens , “Epithelial‐Mesenchymal Transition (EMT) as a Therapeutic Target,” Cells, Tissues, Organs 211, no. 2 (2021): 157–182, 10.1159/000512218. 33401271

[4] G. Manfioletti and M. Fedele , “Epithelial–Mesenchymal Transition (EMT),” International Journal of Molecular Sciences 24, no. 14 (2023): 11386, https://www.mdpi.com/1422‐0067/24/14/11386. 37511145 10.3390/ijms241411386 PMC10379270

[5] Z. Niknami , A. Muhammadnejad , A. Ebrahimi , Z. Harsani , and R. Shirkoohi , “Significance of E‐Cadherin and Vimentin as Epithelial‐Mesenchymal Transition Markers in Colorectal Carcinoma Prognosis,” EXCLI Journal 19 (2020): 917–926, 10.17179/excli2020-1946. 32665775 PMC7355153

[6] S. Y. Chaw , A. Abdul Majeed , A. J. Dalley , A. Chan , S. Stein , and C. S. Farah , “Epithelial to Mesenchymal Transition (EMT) Biomarkers – E‐Cadherin, Beta‐Catenin, APC and Vimentin – In Oral Squamous Cell Carcinogenesis and Transformation,” Oral Oncology 48, no. 10 (2012): 997–1006, 10.1016/j.oraloncology.2012.05.011. 22704062

[7] D. Ribatti , R. Tamma , and T. Annese , “Epithelial‐Mesenchymal Transition in Cancer: A Historical Overview,” Translational Oncology 13, no. 6 (2020): 100773, 10.1016/j.tranon.2020.100773. 32334405 PMC7182759

[8] T. Menju and H. Date , “Lung Cancer and Epithelial‐Mesenchymal Transition,” General Thoracic and Cardiovascular Surgery 69, no. 5 (2021): 781–789, 10.1007/s11748-021-01595-4. 33754237

[9] S. A. Almatroodi , F. A. Alhumaydhi , A. Y. Babiker , A. A. Khan , and A. H. Rahmani , “Potential Therapeutic Targets of Resveratrol, a Plant Polyphenol, and Its Role in the Therapy of Various Types of Cancer,” Molecules 27, no. 9 (2022), 10.3390/molecules27092665. PMC9101422 35566016

[10] K. Guo , Y. Feng , X. Zheng , et al., “Resveratrol and Its Analogs: Potent Agents to Reverse Epithelial‐To‐Mesenchymal Transition in Tumors [Review],” Frontiers in Oncology 11, no. 2021 (2021), 10.3389/fonc.2021.644134. PMC8085503 33937049

[11] Y. Pei , S. Gong , M. Song , A. F. El‐kott , M. Z. Bani‐Fwaz , and Y. Xu , “In Silico Studies, Biological Effects and Anti‐Lung Cancer Potential of Triacetyl Resveratrol as Natural Phenolic Compound,” ChemistrySelect 7, no. 30 (2022): e202201491, 10.1002/slct.202201491.

[12] X. Wang , Y. Liu , K. Li , M. Yang , Q. Wang , and Z. Hao , “Triacetyl Resveratrol Inhibits PEDV by Inducing the Early Apoptosis In Vitro,” International Journal of Molecular Sciences 23, no. 23 (2022): 14499, https://www.mdpi.com/1422‐0067/23/23/14499. 36498827 10.3390/ijms232314499 PMC9737061

[13] M. Thuluz Meza , J.‐P. Claudia , and C. Z. Rossana , “Past, Present, and Future of Molecular Docking,” in Drug Discovery and Development ‐ New Advances, ed. G. Vishwanath , K. Partha , and T. Ashit (IntechOpen, 2020), 10.5772/intechopen.90921.

[14] J. Mazières , W. Brugger , F. Cappuzzo , et al., “Evaluation of EGFR Protein Expression by Immunohistochemistry Using H‐Score and the Magnification Rule: Re‐Analysis of the SATURN Study,” Lung Cancer 82, no. 2 (2013): 231–237, 10.1016/j.lungcan.2013.07.016. 23972450

[15] https://pubchem.ncbi.nlm.nih.gov/.

[16] https://www.rcsb.org/.

[17] M. F. Sanner , “Python: A Programming Language for Software Integration and Development,” Journal of Molecular Graphics & Modelling 17, no. 1 (1999): 57–61. 10660911

[18] C. G. Ricci and P. A. Netz , “Docking Studies on DNA‐Ligand Interactions: Building and Application of a Protocol to Identify the Binding Mode,” Journal of Chemical Information and Modeling 49, no. 8 (2009): 1925–1935, 10.1021/ci9001537. 19655805

[19] R. R. Nasab , F. Hassanzadeh , G. A. Khodarahmi , et al., “Docking Study, Synthesis and Antimicrobial Evaluation of Some Novel 4‐Anilinoquinazoline Derivatives,” Results in Pharma Sciences 12, no. 5 (2017): 425–433, 10.4103/1735-5362.213988. PMC5615873 28974981

[20] M. T. Husunet , R. Mısırlı , E. S. Istıflı , and H. B. Ila , “Investigation of the Genotoxic Effects of Patent Blue V (E131) in Human Peripheral Lymphocytes and In Silico Molecular Docking,” Drug and Chemical Toxicology 45, no. 4 (2022): 1780–1786, 10.1080/01480545.2021.1878208. 33504216

[21] M. Zhang , J. Xue , X. Chen , et al., “Bioactivity of Hamamelitannin, Flavokawain A, and Triacetyl Resveratrol as Natural Compounds: Molecular Docking Study, Anticolon Cancer, and Anti‐Alzheimer Potentials,” Biotechnology and Applied Biochemistry 70, no. 2 (2023): 730–745, 10.1002/bab.2394. 35933706

[22] S. T. Buckley , S. M. Fischer , G. Fricker , and M. Brandl , “In Vitro Models to Evaluate the Permeability of Poorly Soluble Drug Entities: Challenges and Perspectives,” European Journal of Pharmaceutical Sciences 45, no. 3 (2012): 235–250, 10.1016/j.ejps.2011.12.007. 22178532

[23] A. Mateus , A. Treyer , C. Wegler , M. Karlgren , P. Matsson , and P. Artursson , “Intracellular Drug Bioavailability: A New Predictor of System Dependent Drug Disposition,” Scientific Reports 7, no. 1 (2017): 43047, 10.1038/srep43047. 28225057 PMC5320532

[24] F. Lüönd , N. Sugiyama , R. Bill , et al., “Distinct Contributions of Partial and Full EMT to Breast Cancer Malignancy,” Developmental Cell 56, no. 23 (2021): 3203–3221.e3211, 10.1016/j.devcel.2021.11.006. 34847378

[25] J. Fu , A. Shrivastava , S. K. Shrivastava , R. K. Srivastava , and S. Shankar , “Triacetyl Resveratrol Upregulates miRNA‐200 and Suppresses the Shh Pathway in Pancreatic Cancer: A Potential Therapeutic Agent,” International Journal of Oncology 54, no. 4 (2019): 1306–1316, 10.3892/ijo.2019.4700. 30720134

[26] X. Chen , Y. Wang , J. Tian , et al., “Quantitative Chemical Proteomics Reveals Resveratrol Inhibition of A549 Cell Migration Through Binding Multiple Targets to Regulate Cytoskeletal Remodeling and Suppress EMT [Original Research],” Frontiers in Pharmacology 12, no. 2021 (2021), 10.3389/fphar.2021.636213. PMC8044895 33867987

[27] H. Luo , A. Yang , B. A. Schulte , M. J. Wargovich , and G. Y. Wang , “Resveratrol Induces Premature Senescence in Lung Cancer Cells via ROS‐Mediated DNA Damage,” PLoS One 8, no. 3 (2013): e60065, 10.1371/journal.pone.0060065. 23533664 PMC3606183

[28] J. C. Wong , R. Coffman , H. Rosenberg , and R. R. Fiscus , “Abstract 1008: Resveratrol Causes Biphasic Apoptotic and Proliferative Effects and at Higher/Proapoptotic/Anti‐Angiogenesis Concentrations Causes Suppression of Nitric Oxide/cGMP/Protein Kinase G Signaling and Decreased Expression of Prosurvival Proteins c‐IAP1, c‐IAP2, Livin and XIAP in Human Umbilical Vein Endothelial Cells,” Cancer Research 74, no. 19_Supplement (2014): 1008, 10.1158/1538-7445.Am2014-1008.

[29] H. Wang , H. Zhang , L. Tang , et al., “Resveratrol Inhibits TGF‐β1‐Induced Epithelial‐To‐Mesenchymal Transition and Suppresses Lung Cancer Invasion and Metastasis,” Toxicology 303 (2013): 139–146, 10.1016/j.tox.2012.09.017. 23146760

[30] S. Shityakov and C. Förster , “In Silico Predictive Model to Determine Vector‐Mediated Transport Properties for the Blood‐Brain Barrier Choline Transporter,” Advances in Applied Bioinformatics and Chemistry 7 (2014): 23–36, 10.2147/aabc.S63749. PMC4159400 25214795

